# A Case of Sudden Death in the Last Month of Pregnancy

**Published:** 1812-03

**Authors:** J. Ramsbotham


*For the Medical and Physical Journal.*

### A CASE *of* SUDDEN DEATH *in the* LAST MONTH *of* PREGNANCY.

*Communicated by* J. RAMSBOTHAM, M.D.

MRS. M., æt. 33, a tall well-made woman, in her first pregnancy, and within a fortnight of her confinement, was engaged in a party of friends on new-year's evening: during the time she was playing at cards in a heated room, she complained of a disposition to fainting, and immediately withdrew; she walked up stairs into a lodging-room without assistance, seated herself in a chair, very soon fell forward on her knees, uttered a loud shriek, and within a few minutes expired.

Her lifeless situation was at first supposed by her surrounding friends to be a state of syncope; but, its continuance causing alarm, and no symptoms of recovery soon appearing, medical assistance was sent for. The above occurrences took place a few minutes after twelve o'clock. Half an hour or more is supposed to have elapsed before her medical friend arrived, who pronounced the lady to be dead. The consternation of the family at this intelligence, induced them to call in another gentleman in the neighbourhood, and to dispatch a messenger for me. Upon my arrival at the melancholy scene, about half after one, I was made acquainted with the above particulars; and, upon consulting with the gentlemen present, it was considered too late to attempt any thing; the mother had been irrecoverably lost an hour and a half, and it was probable that the infant had already shared the same fate. The lady had been all the previous part of the evening in uncommon spirits. Leave was obtained to inspect the body the next day, but a satisfactory cause of so sudden death was by no means ascertained, unless it can be supposed that the unusual appearances about the uterus were the effect of a cause operating that regretted event.

In the cavity of the abdomen, all the respective viscera were healthy and sound to the eye, except the gravid uterus, which had a suffused black appearance over the greater part of its surface, conveying to my mind the idea of effusion within its parietes under the peritonæal coat, from the rupture of some large vessel. The uterine vessels passing through the broad ligaments were gorged with blood; the Fallopian tubes were turgid and black; and the ovaries, natural in size, had a singular striated or speckled appearance, somewhat like mottled soap. Upon dividing into the substance of the uterus on its anterior part, the placenta was found attached there; but the black suffused appearance above mentioned was more extensive about the fundus and posterior part, where the placenta was not attached. Upon making an incision into the posterior part, the blood followed the knife freely. The child was placed in a natural situation, with the head downward, and was perfectly well grown. The stomach and intestinal canal were somewhat distended, but healthy; and the arch of the colon contained a quantity of fœces.

In the chest, the heart and large blood-vessels were sound; a small quantity of fluid was contained within the pericardium; the right lung was a little diseased with adhesion to the pleura costalis; the left lung was perfectly healthy. The head was not permitted to be examined.

Life, at best, is held by so precarious a tenure, that instances of sudden death are frequent; and, however melancholy the reflexion, such occasional instances seem inseparably attached to the very structure and functions of the human frame. Sudden death is, however, a rare occurrence during a state of pregnancy; for, though such amazing changes changes are effected within the uterine parietes, and upon the abdominal and pelvic viscera and vessels during the process of gestation, Providence has so wisely and effectually counteracted their influence, that they are seldom attended with lasting inconvenience; the process will even go on to its ultimate period under very great disadvantages. When such an event does occur, it makes a strong and injurious impression upon the minds of those pregnant women who are made acquainted with it, so as occasionally to produce serious consequences.

I feel persuaded that, in the present instance, no medical means, however early resorted to, could have saved the mother; the lamp of life was extinguished before any one was aware of it; but I think it probable that the child might have been preserved. The only chance of its preservation was in an immediate resort to the Cæsarean section. That operation has been recommended by authors under different circumstances; but, I apprehend, under no situation whatever, can it be more imperiously demanded as an act of duty on the part of the medical attendant, than in a case of sudden death of the mother in a very advanced stage of pregnancy, with the view of offering a chance of life to the child. With this intention, one important point ought previously to be established; within what lapse of time after the decease of the mother ought it to be performed, to give a chance of life to the infant? How long is it probable a child *in utero* will survive the death of its mother? Various opinions have been held on this question, but I am not aware that it has been at all satisfactorily decided; and I now regret that I did not more strenuously recommend the operation in the present instance, if it had merely gone to the point of correctly ascertaining, whether the circulation in the child had actually ceased or not, within the space of one hour and half. It is commonly supposed that the fœtus *in utero* ceases to live almost immediately upon the death of the mother; but I think it not unreasonable to presume, that, under circumstances like the present, the child's system may be so far perfected, as to allow of the circulation being carried on for some short time after its mother's death; but how long I have no means of judging.* The sooner, however, such an

* I think it also probable, that, even after the lapse of a short time after the circulation in the child has positively ceased, it may possibly be recovered by those means appropriate to the restoration of suspended animation, and especially by complete immersion in warm water, except the face. Such an idea will stimulate our exertions for a reasonable time.

operation operation can be performed after the death of the mother is positively ascertained, the greater chance will there be of saving the infant: of that fact no doubt ought to remain upon the mind. The performance of the Cæsarean section upon a woman who has but just, as it were, expired, seems to carry with it the appearance of cruelty and rashness; but, when it is considered that the mother is insensible to the infliction of pain, that, being deprived of life, it can be of no moment to her, whether her body be opened within a few minutes after her decease or a few hours, and that a very short delay may defeat the very object in view, which is a chance of preservation to the child, the idea of cruelty and rashness will be lost in a sense of duty.

The prejudices of surrounding friends will not be easily surmounted; their reluctance to accede to an operation apparently so dreadful, will rise superior to the considerations of duty, or to the value of the infant's life; yet every argument ought to be used to shew the weakness and impropriety of such conduct; to induce a ready acquiescence in the means proposed, as the only chance of preserving the infant.

In performing the Cæsarean section in such a case, before an opening be made into the cavity of the uterus, it would be desirable to ascertain at what part the placenta is attached, that it may not be unnecessarily divided, and the blood of the child, if the circulation be going on, be discharged. This observation naturally occurs from the situation of the placenta in the present case; which, being at the anterior part of the uterus, was cut into, on opening that viscus, before the operator was aware.

In thus offering my sentiments upon the propriety, nay necessity, of having immediate resource to the Cæsarean section, in case of sudden death of the mother, I by no means wish to be considered an advocate for that operation during her life; its fatality has been so general in this country, that I could never propose it with any idea of saving the mother. That operation appears to me only justifiable in a case of sudden death of the mother in the last month of pregnancy; or in one in which there is a high degree of deformity in the pelvis, with a preternatural presentation, and at the same time where the woman is bowed down with disease, so as to make her life of little relative value. A case of this kind, in which the operation was performed, the child was taken out of the uterus living, though it afterwards died, and the mother died also the succeeding day, is related by Dr. Kellie, in the Edinburgh Medical Journal for this month, (January, 1812.)

